# "Seeing" the Difference: The Importance of Visibility and Action as a Mark of "Authenticity" in Co-production: Comment on "Collaboration and Co-production of Knowledge in Healthcare: Opportunities and Challenges"

**DOI:** 10.15171/ijhpm.2016.136

**Published:** 2016-10-17

**Authors:** Jo Cooke, Joe Langley, Dan Wolstenholme, Susan Hampshaw

**Affiliations:** ^1^NIHR Collaboration and Leadership in Applied Health Research and Care for Yorkshire and Humber (CLAHRC YH), Sheffield, UK.; ^2^Lab4Living, Sheffield Hallam University, Sheffield, UK.; ^3^Translating Knowledge Into Action, NIHR CLAHRC Yorkshire and Humber, Sheffield, UK.; ^4^Doncaster Metropolitan Borough Council, Doncaster, UK.

**Keywords:** Co-production, Knowledge Mobilisation, Design Approaches in Healthcare, Research Impact, Actionable Tools

## Abstract

The Rycroft-Malone paper states that co-production relies on ‘authentic’ collaboration as a context for action. Our commentary supports and extends this assertion. We suggest that ‘authentic’ co-production involves processes where participants can ‘see’ the difference that they have made within the project and beyond. We provide examples including: the use of design in health projects which seek to address power issues and make contributions visible through iteration and prototyping; and the development of ‘actionable outputs’ from research that are the physical embodiment of co-production. Finally, we highlight the elements of the Collaboration for Leadership in Applied Health Research and Care (CLAHRC) architecture that enables the inclusion of such collaborative techniques that demonstrate visible co-production. We reinforce the notion that maintaining collaboration requires time, flexible resources, blurring of knowledge producer-user boundaries, and leaders who promote epistemological tolerance and methodological exploration.

We read with interest the paper by Rycroft-Malone and colleagues.^1^ The conclusions drawn from the paper resonates with our own experiences as applied researchers. It confirms the importance of blurring the boundaries between knowledge creation and knowledge use through integrating multiple stakeholders’ perspectives in research and implementation activity. It also supports the notion that such approaches should be iterative and incremental. The paper suggests that co-production relies on ‘authentic collaboration, partnership and engagement as the context for action’ (p 221). It is the nature of both authenticity and impact linked to action that we would like to explore further in this commentary, drawing on our experiences of working in a Collaboration for Leadership in Applied Health Research and Care (CLAHRC). We will argue that there are distinctive qualities in co-production that makes iterations within the process, and outcomes visible.

Understanding context is essential in relation to how mechanisms of engagement have an impact on health services.^2^ Our commentary is based on reflections of working in a collaboration that defined co-production as engaging ‘the right people’ (service users, practitioners, NHS and care managers, and academics from a range of disciplines) to make decisions and support the conduct of projects and activities on issues that are important and matter to them.^3^ We would suggest that co-production should seek to *make visible* the *activities* that mobilise and coproduce knowledge and the *coproduced knowledge* itself, throughout and beyond the project, thus, exemplifying the shared nature of meaningful and authentic collaboration.

In building this argument, we will explore a number of issues: Firstly, to illustrate methodologies that are mindful of power issues that promote co-production of knowledge. We will focus here on projects that include service users amongst other stakeholders, with particular reference to the use of *design* in health projects. We will then explore how making ‘objects’ that are the physical embodiment of co-production, offers opportunities for visible impact within the project and beyond. Finally, we will describe the elements of the CLAHRC architecture that enable the inclusion of these co-production techniques.

## Methodologies That Support Co-production and Address Power Relations

Co-production is increasingly described as a method of addressing power imbalances for designing and delivering impact on public services.^4^ The Rycroft-Malone paper recognizes that knowledge user communities are many and varied. Such partnerships include researchers and practitioners^5^ and more recently patient and public involvement (PPI).^6^ Many challenges are experienced in partnerships that include diverse groups of stakeholders.^7^ Each group has their own cultural norms and language, which often reinforces hierarchy.^8^ A recent narrative literature review^9^ of PPI in healthcare improvement concluded that PPI models are currently too narrow, and invite us to look for alternative approaches which focus on empowerment of participants. They put forward a number of principles that support an empowering approach including recognising peoples’ assets, building on participants’ strengths, blurring of professional and patient boundaries, networking, and supporting a shift from delivering services to supporting things to happen. We propose that the use of design methods in health projects is such an approach, as it has a value base that aligns to these principles.Additionally, design seeks to change the world^10-13^ rather than study how it is. This primarily includes ‘making things’, but also embraces ‘making things happen’ which is an important motivation for PPI.^7^

Design methods address power relations because the approach blurs distinctions between ‘knowledge user’ and ‘knowledge producer’ communities, and harness activity where ‘*knowledge is the individual ability to draw distinctions within a collective domain of action’*^9^ thereby recognizing assets and building on strengths of each participant. It achieves this through the process of making. Making is not only the end product of the design process but it is the means by which designers and participants can create new meaning and knowledge ‘through’ sketching, simple prototyping, or other creative practices. It includes a systematic approach to inclusivity, because it assists each individual to develop their own thoughts through the act of making, and then collectively, to arrive at shared understandings. Making of things is not the sole preserve of stakeholders who usually hold more power, and all have input into the end product.

Besides mediating social dynamics within a diverse group, the making of artefacts has other qualities. Instead of trying to find a verbal language, creative approaches can support participants to ‘think with their hands’ and so addresses issues of power mediated through language which is often a difficulty when bringing such diverse groups together.^14^ It also enables participants to make things together, which promotes engagement and inclusion^15,16^ Our experience, and those of other authors^17^ is that the process itself can be impactful, and can create products that are immediately useful. We would like to illustrate this through two examples: one linked to ‘making things,’ and another to ‘making things happen.’

The development of Mental Capacity Assessment Support Toolkit (MCAST) is an example of a co-design approach to ‘making things.’ This toolkit aims to test the difference between barriers to communication and mental incapacity in patients, and gives the professional a range of communication techniques to allow them to make an assessment in the event of communication challenges.^18^ An initial outline prototype of the MCAST was developed based on a theoretical framework derived from literature with expert professional input, followed by co-design workshops with service users and practitioners. These workshops explored physical layouts that resulted in further toolkit iterations, which were shared with the stakeholders leading to a final product. Photographs to aid communication were an important part of the toolkit. These were developed with service users, who selected the final examples that appear in the toolkit. Stakeholders commented that seeing their previous advice embodied within the iterations of the prototypes made visible their contribution, and demonstrated that what they were suggesting was being listened to and acted upon in a tangible way. The final product is now being tested out within National Health Service (NHS) organizations. We are finding that the photograph element of the toolkit is being used for other activities to assist communication, as well as for assessment proposes, which demonstrates their usefulness for broader communication. Similar design activities have been used to develop other actionable outputs (see below), and in coproducing medical technology solutions (see http://www.lab4living.org.uk/) to show visible and authentic collaboration.

Another design project helped to ‘make things happen’, and used the Better Services by Design approach (http://www.bsbd.org.uk/) in a public health intervention through community participation. The project focused on the use of a lakeside space next to a retail park to increase physical activity. The project brought together Public Health practitioners with a diverse range of stakeholders, including the local football team, retail providers, nature groups, tourism and residents, to plan how to make the lakeside accessible to all. Views were sought through social media, surveys and photography undertaken by community participants as they used the space. Often views about how to increase the use of the lakeside were conflicting. The design approach included a ‘walking together’ workshop and used the knowledge sources described earlier to develop a joint solution which included a milestone/way finder architecture which could be used by all the stakeholders to include heritage trails, nature walks, and family treasure hunts. The milestones included step counts to measure activity. This activity has now been implemented (http://www.doncaster.gov.uk/services/culture-leisure-tourism/discover-lakeside), and proposals are in place to evaluate the effectiveness of the intervention in respect to increased exercise and activity. The council highlighted, that whilst the milestone architecture could have been developed by council officials, the joint ownership through its development, reduced conflicting views and enabled community engagement.

## Actionable Outputs and Tangible Returns

Another way to make visible, authentic collaboration is through the co-production of research-derived ‘actionable tools’ that are useful to policy or practice. We believe this can be beneficial to both research and practice partners, and is a useful adjunct to the peer reviewed paper as legitimate academic output which has been defined as problematic for some stakeholders in co-production partnerships.^19^

Rycroft-Malone and colleagues strongly support the need for research-practice collaborative partnerships that align stakeholder priorities and motivations in order to address ‘what’s in it’ for each. We would suggest that a fruitful area for consideration is the co-production of outputs or tools from research projects developed to address real-world problems. We call these ‘actionable tools.’ These are products informed by a research study’s findings that are intended to change the way of thinking, promote decision-making, or instigate action around an issue. As such they can be considered as boundary objects ‘that enhance the capacity of an idea, theory or practice to translate across culturally defined boundaries.’^20^ These tools can focus on influencing patients, practice and healthcare systems, and examples include commissioning tools, teaching and learning packs, patient decision aids and patient reported outcome measures. Whilst some authors^21,22^ have found that boundary objects can become a barrier to change if they continue to reinforce power structures, we have found that if they are co-produced, they are more likely to be impactful and fit for purpose. Such an approach supports the notion of integrated knowledge transfer^23^ and we have developed such outputs using design and other participatory approaches. The co-production of such outputs also ‘blurs’ the boundaries between knowledge users and producers described by Evans and Scarborough,^24^ which can overcome issues described where research teams and implementation teams are kept separate.^22^

Whilst Kislov^22^ comments that boundary objects emerging from within communities of practice have not, as yet been tested, we think this looks promising as exemplified by some developing case studies (http://clahrc-yh.nihr.ac.uk/resources/case_study_library). Such tools can demonstrate reciprocity across stakeholder groups. For example, one project developed pen portraits of older people (http://kwillt.org/index.php/products) who are at risk of being cold at home.^25^ Pen portraits are descriptive narratives to explain health behaviours in complex environments, and were developed in order to inform targeted interventions in policy and practice. These were coproduced with practitioners in the field who then used them for local policy, but they were also used nationally for the cold weather plan in England. However, this was also recognized in academia as an example of good practice by the Council of Deans in the Research Excellence Framework (http://www.councilofdeans.org.uk/wp-content/uploads/2014/12/Care-Transformed-web-version-1.pdf) and was, therefore, beneficial to academic partners also.

## Flexible Architecture That Accommodates Flexible Authentic and Visible Co-production

Certain elements of the CLAHRC infrastructure enable co-production techniques. These include distributed funding and leadership models in a long term funded partnership, a remit to conduct research and implementation with demonstrable impact in a short timeframe, and a unique matched funding model with service providers.

CLAHRCs are funded by the NIHR for five-year periods to create a distributed model for the conduct and application of applied health research that links research and practice.^26^ Such lengthy funding enables the ‘softer’ aspects such as relationship building suggested by Rycroft-Malone et al. CLAHRCs also have a remit to undertake research *and* knowledge mobilization which promotes non-linear understandings of the research-practice relationship that supports co-production^27,28^ that promote action (and therefore, authenticity) when twinned with distributed leadership^29^ and, in turn, a flexible resource to do this.^30^

The CLAHRC unique envelope includes funding from the NIHR, which is ‘matched’ pound for pound by partners. In this way, the matched funding represents a blurring of the boundaries between research funders, research producers, and research users. Matched funding usually involves practitioner and policy-makers’ time to get involved in the collaboration, and ‘legitimises’ time to undertake co-production activity. However, services with a limited resource are unlikely to offer contributions unless they see benefit in doing so, thus, accountability for visible impact is built in to the collaboration. Our experience is that matched funding activity supports both solution focused design approaches, and actionable tools development, as both are perceived as useful and impactful.

As the Rycroft-Malone article suggests the co-production approach is not a panacea. We would reinforce the notion that maintaining collaboration requires time, tolerance and a form of leadership that involves navigation around difficulties, and promotes an environment of epistemological tolerance. However, not all academics or practitioners are comfortable in working in such environments. Engaging with such diverse disciplines and techniques such as those described in this commentary involves insightful and sometimes courageous leadership and engagement, at many levels, and throughout the collaboration.

## Acknowledgments

The research was funded by the NIHR CLAHRC Yorkshire and Humber, Sheffield, UK (http://clahrc-yh.nihr.ac.uk/). The views expressed are those of the author(s), and not necessarily those of the NHS, the NIHR or the Department of Health.

## Ethical issues

Not applicable.

## Competing interests

Authors declare that they have no competing interests.

## Authors’ contributions

JC was the lead author. JL, DW, and SH provided key input on case studies. All authors revised and finalized it.

## Authors’ affiliations

^1^NIHR Collaboration and Leadership in Applied Health Research and Care for Yorkshire and Humber (CLAHRC YH), Sheffield, UK. ^2^Lab4Living, Sheffield Hallam University, Sheffield, UK. ^3^Translating Knowledge Into Action, NIHR CLAHRC Yorkshire and Humber, Sheffield, UK. ^4^Doncaster Metropolitan Borough Council, Doncaster, UK.

## References

[1] Rycroft-Malone J, Burton CR, Bucknall T, Graham ID, Hutchinson AM, Stacey D (2016). Collaboration and co-production of knowledge in healthcare: opportunities and challenges. Int J Health Policy Manag.

[2] Boaz A, Hanney S, Jones T, Soper B (2015). Does the engagement of clinicians and organisations in research improve healthcare performance: a three-stage review. BMJ Open.

[3] Cooke J, Ariss S, Smith C (2015). On-going collaborative priority-setting for research activity: a method of capacity building to reduce the research-practice translational gap. Health Res Policy Syst.

[4] Boyle D, Slay J, Stephens L. Public services inside out. Putting co-production into practice. London: NESTA; 2010.

[5] McCabe K, Wallace A, Crosland A (2015). A model for collaborative working to facilitate knowledge mobilisation in public health. Evid Policy.

[6] Wilson P, Mathie E (2016). Research with patient and public involvement: a realist evaluation – the RAPPORT study. Health Serv Deliv Res.

[7] Ocloo J, Matthews R (2016). From tokenism to empowerment: progressing patient and public involvement in healthcare improvement. BMJ Qual Saf.

[8] Robinson K. Out of our Minds. Capstone Publishing; 2011.

[9] Tsoukas H, Vladimirou E (2001). What is Organisational Knowledge?. J Manage Stud.

[10] Cross N. Designerly Ways of Knowing. London: Springer-Verlag; 2006.

[11] Gregory S A. The Design Method. London: Butterworth; 1966.

[12] March LJ. The logic of design and the question of value. The Architecture of Form. UK: Cambridge University Press; 1976.

[13] Simon HA. The Sciences of the Artificial. Cambridge: MIT Press; 1969.

[14] Hinchcliff R, Greenfield D, Braithwaite J (2014). Is it worth engaging in multi-stakeholder health services research collaborations? Reflections on key benefits, challenges and enabling mechanisms. Int J Qual Health Care.

[15] Gauntlett D. Making is connecting; the social meaning of creativity, from DIY and knitting to YouTube and Web 2. London: Polity Press; 2011.

[16] Kontos PC, Poland BD (2009). Mapping new theoretical and methodological terrain for knowledge translation: contributions from critical realism and the arts. Implement Sci.

[17] Vink J, Wetter-Edman K, Edvardsson B, Tronvol B. Understanding the influence of the co-design process on well-being. Paper presented at: Serv Des; May 24-26, 2016; Copenhagen.

[18] Jayes M, Palmer R, Enderby P (2015). Development of a mental capacity assessment support tool: a user-centred design process. Int J Stroke.

[19] Gillard S, Simons L, Turner K, Lucock M, Edwards C (2012). Patient and public involvement in the co-production of knowledge: reflection on the analysis of qualitative data in a mental health study. Qual Health Res.

[20] Fox NJ (2011). Boundary objects, social meanings and the success of new technologies. Sociology.

[21] Oswick C, Robertson M (2009). Boundary objects reconsidered: from bridges and anchors to barricades and mazes. Journal of Change Management.

[22] Kislov R (2014). Boundary discontinuity in a constellation of interconnected practices. Public Adm.

[23] McGrath OC, Lingley-Pottie P, Johnson Emberly D, Thurston C, McLean C (2009). Integrated Knowledge Translation in Mental Health: Family Help as an Example. J Can Acad Child Adolesc Psychiatry.

[24] Evans S, Scarbrough H (2014). Supporting knowledge translation through collaborative translational research initiatives: ‘Bridging’ versus ‘blurring’ boundary-spanning approaches in the UK CLAHRC initiative. Soc Sci Med.

[25] Tod A, Lusambili A, Homer C, Abbott J, Cooke J, McDaid K. Understanding factors influencing vulnerable older people keeping warm and well in winter: a qualitative study using social marketing techniques. BMJ Open. 2012;2(4). pii: e000922. 10.1136/bmjopen-2012-000922 PMC3400059 22798252

[26] Best research for best health, implementation plan. NIHR; 2009.

[27] Martin GP, Ward V, Hendy J (2011). The challenges of
evaluating large-scale, multi-partner programmes: the
case of NIHR CLAHRCs. Evid Policy.

[28] Walshe K, Davies HT (2013). Health research, development and innovation in England from 1988 to 2013: from research production to knowledge mobilization. J Health Serv Res Policy.

[29] D’Andreta D, Scarbrough H, Evans S (2013). The enactment of knowledge translation: a study of the collaborations for leadership in applied health research and care initiative within the English National Health Service. J Health Serv Res Policy.

[30] Currie G, Lockett A, El Enany N (2013). From what we know to what we do: lessons learned from the translational CLAHRC initiative in England. J Health Serv Res Policy.

